# No differences in clinical outcomes between fixed- and mobile-bearing computer-assisted total knee arthroplasties and no correlations between navigation data and clinical scores

**DOI:** 10.1007/s00167-014-3127-x

**Published:** 2014-06-15

**Authors:** Carlos J. Marques, Sandra Daniel, Anusch Sufi-Siavach, Frank Lampe

**Affiliations:** 1 Research Center of the Orthopedic and Joint Replacement Department, Schoen Klinik Hamburg Eilbek, Dehnhaide 120, 22081 Hamburg, Germany; 2 Orthopedic and Joint Replacement Department, Schoen Klinik Hamburg Eilbek, Dehnhaide 120, 22081 Hamburg, Germany; 3 Faculty of Life Sciences, Hamburg University of Applied Sciences, Lohbrügger Kirchstraße 65, 21033 Hamburg, Germany

**Keywords:** Mobile bearing, Fixed bearing, Total knee replacement, Computer-assisted surgery, Randomized controlled trial

## Abstract

**Purpose:**

The theoretical advantages of mobile-bearing (MB) designs over the conventional fixed bearings (FBs) for total knee arthroplasty (TKA) have not been proved yet through clinical studies. The aim of the study was to test whether the MB design has advantages in terms of better clinical outcomes when compared to FB. Furthermore, the relationships between intra-operative obtained implant positioning data and the clinical scores were analysed.

**Methods:**

A total of 99 patients were randomized into the FB or the MB group. All patients received the same posterior cruciate retaining implants and were operated with the use of a computer-assisted navigation system. The clinical outcomes of both groups were compared pre-operatively, at 1 year, and at a mean follow-up time of 4 years after surgery.

**Results:**

The MB implants showed no advantages over the FB when comparing the Knee Society Scores, the Oxford Score, the range of movement (ROM) and pain intensity of the patients in both groups at 1 and 4 years after surgery. There were no relationships between the computer navigation data and the clinical scores.

**Conclusions:**

In view of the 4-year results, there is no evidence to support the recommendation of one design over the other in terms of better clinical outcome scores, higher ROM or lower pain rates. Long-term follow-up results may be necessary, including survival rates. Further research comparing different TKA designs should also include standardized performance-based tests.

**Level of evidence:**

Prospective study (Randomized controlled trial with adequate statistical power to detect differences), Level I.

## Introduction

Patients submitted to total knee arthroplasty (TKA) experience a significant improvement in their health-related quality of life (HRQOL) already soon after surgery [2, 4, 8, 27]. The HRQOL improvements observed at short term maintain over the years [8, 19, 26].

In observational studies, the long-term survival rates reported after TKA are high and range from 90 % at 15 years [30] to 82 % at 22 years [32]. Despite high survival rates, causes of revisions like short-term anterior knee pain, aseptic loosening of the tibia component and wear of the polyethylene (PE) insert remained unsolved. Attempting to solve these problems, the mobile-bearing (MB) designs were introduced. MB TKA designs were claimed to reduce the risk of aseptic loosening by minimizing the stress transmitted to the prothesis–bone interface, to reduce the wear of the PE insert by increasing the implant conformity [34, 41], to increase the overall range of motion by allowing the femur to roll back during flexion and to rotate during extension and to reduce the anterior knee pain rates [40].

These theoretical advantages of MB over fixed bearing (FB) designs could not be demonstrated by recently published studies [5, 9, 28]. In a systematic review and meta-regression including 41 studies comparing MB versus FB for TKA, the authors found no clinically relevant differences in terms of clinical outcome scores, revision rates and patient-reported outcome measures [37].

To minimize bias that may emerge with the use of different implant types, different surgical techniques and post-operative rehabilitation programs, a randomized double-blind clinical trial was designed. All surgeries were performed with a computer-assisted navigation system by two experienced surgeons, using always the same cruciate retaining implant types. The only varying parameter was the mobility of the PE inlay. The aim of the study was to compare the effects of fixed versus MB in TKA on clinically relevant outcomes. The Knee Society Score (KSS) [16] of both groups was compared as primary end point. The Oxford Score (OXF) [11], the range of movement (ROM—passive flexion) and two sub-items of the KSS (KSS-Pain and KSS-Stairs) were also compared between the groups as secondary outcomes. A secondary purpose of this study was to test for relationships between the intra-operative-obtained computer navigation data and the clinical scores. The following hypotheses were formulated: (1) there would be no differences between the MB and FB groups across the follow-up assessments; and that (2) there would be no significant relationships between the navigation data and the clinical scores.

## Materials and methods

To study potential effects of the implant type on clinical relevant outcomes, a double-blind randomized controlled trial was designed. From April 2004 until June 2007, 99 patients (100 knees) scheduled for primary bicondylar, posterior cruciate retaining TKA at the Schoen Klinik Hamburg Eilbek, Germany, were informed about the study and agreed to participate. Before participating, all patients were required to read and sign an informed consent form.

If the patients met the inclusion criteria (clinical and radiological signs of osteoarthritis of the knee with failed non-operative treatment; no indication for a uni-compartmental implant or joint-preserving osteotomies; age ranging from 40 to 90 years; American society of anaesthesiologists pre-operative classification grade 1–3; no deformity larger than 20 varus or 15° valgus; no previous bone surgery to the index knee; no previous total joint replacement at the index leg; no post-operative infection of the index knee or thrombosis within the follow-up period), they were randomly assigned either to the FB or the MB group. The randomization was made with concealed envelopes labelled with random numbers. Neither the patients nor the assessor knew in which group the patient was allocated (double-blind). Only the surgeon got the information inside the concealed envelope on which kind of implant the patient should get.

At each examination the OXF, the KSS and ROM of the indexed knee were assessed by a trained physician. The patients received standardized instructions and were required to answer the OXF questionnaire on their own. The KSS questionnaire was answered with support of the physician. German translations of both questionnaires were used.

Range of movement was assessed with an analogue goniometer with the patient lying in the supine position as described in the literature [25].

All patients were operated by one of two senior surgeons with the use of an imageless computer navigation system (Orthopilot TKA 4.2, BBraun Aesculap, Tuttlingen, Germany) [15], allowing the acquisition of the following intra-operative implant positioning data: femoral angle coronal (FAC); femoral angle sagittal (FAS); tibial angle coronal (TAC); tibial angle sagittal (TAS).

In the FB group, the implant used (Columbus CR, BBraun Aesculap, Tuttlingen, Germany) had a PE inlay rigidly fixed to the tibial tray. In the MB version of the implant (Columbus RP, BBraun Aesculap, Tuttlingen, Germany), the PE inlay rotates around a cylindrical post within a range of ±10° limited by a second post placed interiorly on the surface of the tray. The femoral components were identical in both groups. All components were cemented, and no patellar components were implanted.

After surgery, all patients followed a standard rehabilitation protocol, including self-controlled epidural analgesia with ropivacaine, non-steroidal oral analgesia and antithrombotics. Physiotherapy started one day after surgery.

During the patient recruitment period, 52 patients were randomly allocated in the FB and 48 in the MB group. The mean age of the patients by entrance in the study was 68.9 ± 8.4 and 69.4 ± 7.1 years for the FB and MB groups, respectively. The mean difference was statistically not significant (n.s.) when comparing the groups. The mean body mass index (BMI) of both groups was also statistically equal (n.s.). For further demographic data, see Table 1.

**Table 1:** Demographic data of the sample by the time of entry in the study

Variables	All	FB	MB	Mean diff. (*p* value) [95 % CI]
Number of patients	*n* = 100	*n* = 52	*n* = 48	
Gender (F; M)	74 F; 26 M	39 F; 13 M	34 F; 14 M	
Age (years)	69.1 ± 7.8	68.9 ± 8.4	69.4 ± 7.1	0.4 (n.s.)
Body weight (kg)	82.6 ± 15.7	79.6 ± 13.8	85.9 ± 17	6.3 (*p* = 0.04) [−12.5 to −0.09]*
Body height (cm)	167.1 ± 8.4	166.4 ± 8.7	168 ± 8	1.6 (n.s.)
BMI (kg/m^2^)	29.5 ± 5.5	28.7 ± 4.9	30.4 ± 6	1.6 (n.s.)

Values are mean ± SD

*FB* fixed bearing, *MB* mobile bearing, *M* male, *F* female, *n.s.* non-significant

* Significant difference

**Fig. 1:**
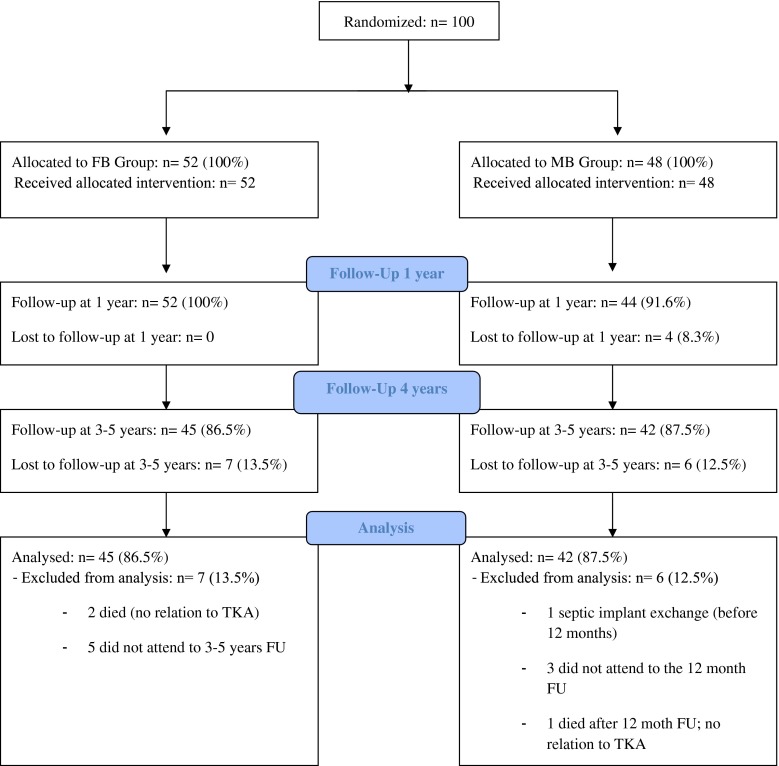
Patient flow diagram according to the CONSORT statement. There is neither available data on the number of patients assessed nor on the number of patients excluded and their exclusion reasons

The patients were followed up at 12 months and at a mean follow-up time of 4 years post-surgery. At 1 year, there was no patient lost to follow-up in the FB group and four patients dropped out in the MB group: one patient was excluded because of a septic implant exchange before 12 months and three did not attend the 12-month follow-up examination. Four years after surgery, there was a lost to follow-up rate of 13.5 (*n* = 7) and 12.5 % (*n* = 6) in the FB and MB groups, respectively. The reasons of lost to follow-up were neither related to surgery nor to the type of implant used and are exposed in the flow diagram (Fig. 1).

The Medical Ethics Commission of the Federal State of Hamburg approved the research proposal (File #2226). The trial was registered under ClinicalTrials.gov (NCT00822640).

The data of 87 patients (45 in the FB and 42 in the MB group) were included for analysis. The normal distribution of the variable data was confirmed with the Kolmogorov–Smirnov test. Independent variable data comparisons between the two groups at baseline were performed with a *t* test for independent samples (Table 1).

A mixed design ANOVA (2 × 3) for repeated measures was chosen to test for differences between the KSS-Function, KSS-Knee, OXFs and ROM (passive flexion) between the groups across the measurement times. The Greenhouser-Geisser correction was chosen when the sphericity assumption was not assumed. In cases where the 2 × 3 ANOVA revealed no significant interactions between the implant type (FB vs. MB) and the repeated measure factor, and no significant main effects for the implant type, a one-way ANOVA was performed for the all sample to test for differences across the time. Post hoc multiple comparisons were made with paired *t* tests with the Bonferroni adjustment of the alpha.

A nonparametric approach was chosen to analyse KSS sub-items “Pain” and “Stairs”, since the values were not normal distributed. To compare the sub-items of both groups at each follow-up time, the Man-Whitney *U* test was used. When no differences were found between the FB and MB groups at each point in time for each of the sub-items, both groups were collapsed and further analysis was made for the all sample. A Friedman’s ANOVA was performed to test for differences between the scores at baseline, 1 year and at 4 years. Multiple comparisons were conducted between the paired follow-up times with Wilcoxon signed rank procedures with alpha set at 0.017 (0.05/3 tests = 0.017) to compensate for alpha inflation with multiple testing.

Pearson’s product-moment correlation (*r*) was run to determine the relationships between the intra-operative obtained navigation data (implant positioning data), leg alignment data and the clinical scores.

Power analysis was performed based on the KSS values reported in the literature [10, 29]. An estimated effect size of *d* = 0.67 with the alpha set at 0.05 and beta set at 0.15 revealed a sample size of 42 patients per group. The sample size was raised up to 50 patients per group, accounting for 20 % lost to follow-up.

All statistical tests were carried out with the use of the IBM SPSS software version 21 for Mac. For all statistical tests, except for multiple comparisons, the 0.05 level of probability was accepted as the criterion for statistical significance.

## Results

Knee Society Score-Function scores were not equal when comparing both groups across time (*F* = 4.2; *p* = 0.02). Post hoc analysis revealed a ten points significant better KSS-Function result for the FB group at baseline (*p* = 0.009). There were neither significant mean KSS-Function differences between both groups at 1 nor at 4 years (Table 2). The mean KSS-Function improvements between pre-operatively and 1 year were 35.4 ± 18.2 and 45 ± 21.1 for the FB and MB groups, respectively. The mean difference was significant (*p* = 0.01), showing a significantly greater post-operative improvement for the patients in the MB group during the first post-surgery year. There was a significant main effect for the repeated measure factor (*p* < 0.001). KSS-Function improved significantly from baseline to 1 year (*p* < 0.001) and stabilized from 1 to 4 years (n.s.) in both groups.

**Table 2:** Data of the dependent variables across the measurement times

	Group	Pre	1 year	4 years	Results
KSS-Function	FB (*n* = 45)	60 (0–70) 53.7 ± 16.8	90 (45–100) 89 ± 13.2	90 (30–100) 85 ± 16.9	2 × 3 ANOVA results: (1) significant interaction between implant type and time (*F* = 4.2; *p* = 0.02)*; post hoc significant mean KSS-F difference between FB and MB pre-operatively: 10.3 (*p* = 0.009) [2.6–18.0]*; (2) significant main effects for the factor time (*F* = 235.9; *p* < 0.001)*; post hoc-FB: pre to 1 year: 35.2 (*p* < 0.001) [−42.1 to −28.2]* 1–4 year: 4.0 (n.s.); MB: pre to 1 year: 45.1 (*p* < 0.001) [−53.3 to −36.8]* 1–4 year: 3.0 (n.s.)
	MB (*n* = 42)	50 (0–70) 42.9 ± 21.4	90 (55–100) 88.1 ± 11.5	90 (60–100) 85 ± 13	
	All (*n* = 87)	60 (0–70) 48.5 ± 19.8	90 (45–100) 88.5 ± 12.4	90 (30–100) 85 ± 15.1	
KSS-Knee	FB	29 (0–56) 29.5 ± 10.8	89 (41–100) 86.9 ± 12.4	89 (40–100) 85.1 ± 13.5	2 × 3 ANOVA results: no significant interaction between implant type and time (*F* = 0.1; n.s.); significant main effects for the factor time (*F* = 815.8; *p* < 0.001)
	MB	25.5 (17–55) 29.4 ± 9.6	91.5 (40–100) 88.1 ± 11.9	89.5 (65–100) 87 ± 9	
	All	28 (0–56) 29.4 ± 10.2	91 (40–100) 87.5 ± 12.1	89 (40–100) 86 ± 11.5	Pre to 1 year: 58 (*p* < 0.001) [−62.5 to −53.5]* 1–4 years: 1.4 (n.s.)
Oxford Score	FB	39.5 (22–55) 39.9 ± 7	17.5 (12–52) 19.6 ± 8.4	16 (12–52) 19.8 ± 9.8	2 × 3 ANOVA results: no significant interaction between implant type and time (*F* = 1.5; n.s.); significant main effects for the factor time (*F* = 345; *p* < 0.001)*
	MB	42 (31–53) 42.5 ± 4.7	18 (12–43) 20.2 ± 7.9	16 (12–44) 19.4 ± 7.5	
	All	41 (22–55) 41.2 ± 6.1	18 (12–52) 19.9 ± 8.1	16 (12–52) 19.6 ± 8.7	Pre to 1 Y: 21.3 (*p* < 0.001) [18.9–23.6]* 1–4 Y: 0.3 (n.s.)

Values are median (range) and mean ± SD for the Knee Society Score (KSS) and Oxford Score

*MB* mobile bearing, *FB* fixed bearing; *Pre* pre-operatively, *Y* Year(s), *n.s.* non-significant, *Y* year(s)

* Significant difference

Knee Society Score-Knee score differences between the groups across the measurement times were not significant (n.s.). Ignoring the implant type, there was a significant main effect for the repeated measure factor (*p* < 0.001). The mean KSS-Knee score improved significantly from baseline to 1 year (*p* < 0.001) and maintained between 1 and 4 years (n.s.).

Oxford Scores of both groups across the time were not significantly different. Ignoring the implant type, there was a significant effect for the repeated measure factor (*p* < 0.001). The OXF improved significantly for all patients between baseline and 1 year (*p* < 0.001) and remained stable between 1 year and the last follow-up.

Range of movement of both groups was not different from each other across the time (Table 3). Once again, ignoring whether the patients got a FB or a MB implant, there was an overall significant difference in ROM across the measurement times (*p* < 0.001). Pairwise comparisons shown that a 3.8 % overall mean ROM increase between baseline and 1 year was statistically significant (*p* = 0.01). ROM remained stable between 1 and 4 years (n.s.).

**Table 3:** Data of the dependent variable ROM (passive flexion)

Group	Pre-operative	1 year	4 years
*ROM*			
FB (*n* = 45)	110.6 ± 15.5	112.8 ± 13.3	114.3 ± 9.3
MB (*n* = 42)	109.4 ± 12.7	115.7 ± 11.1	117.7 ± 10.9
All (*n* = 87)	110 ± 14.2	114.2 ± 12.3	115.9 ± 10.2
Mean diff. (*p* value) [95 % CI] Diff. rates (%)	4.2 (*p* = 0.01) [−7.9 to −0.6]* 3.8 % increase from baseline to 1 year		1.7 (n.s.) 1.4 % increase from 1 to 3–5 years

Values are mean ± SD for range of movement (ROM–passive flexion)

*MB* mobile bearing, *FB* fixed bearing, *n.s.* non-significant

* Significant difference

**Fig. 2:**
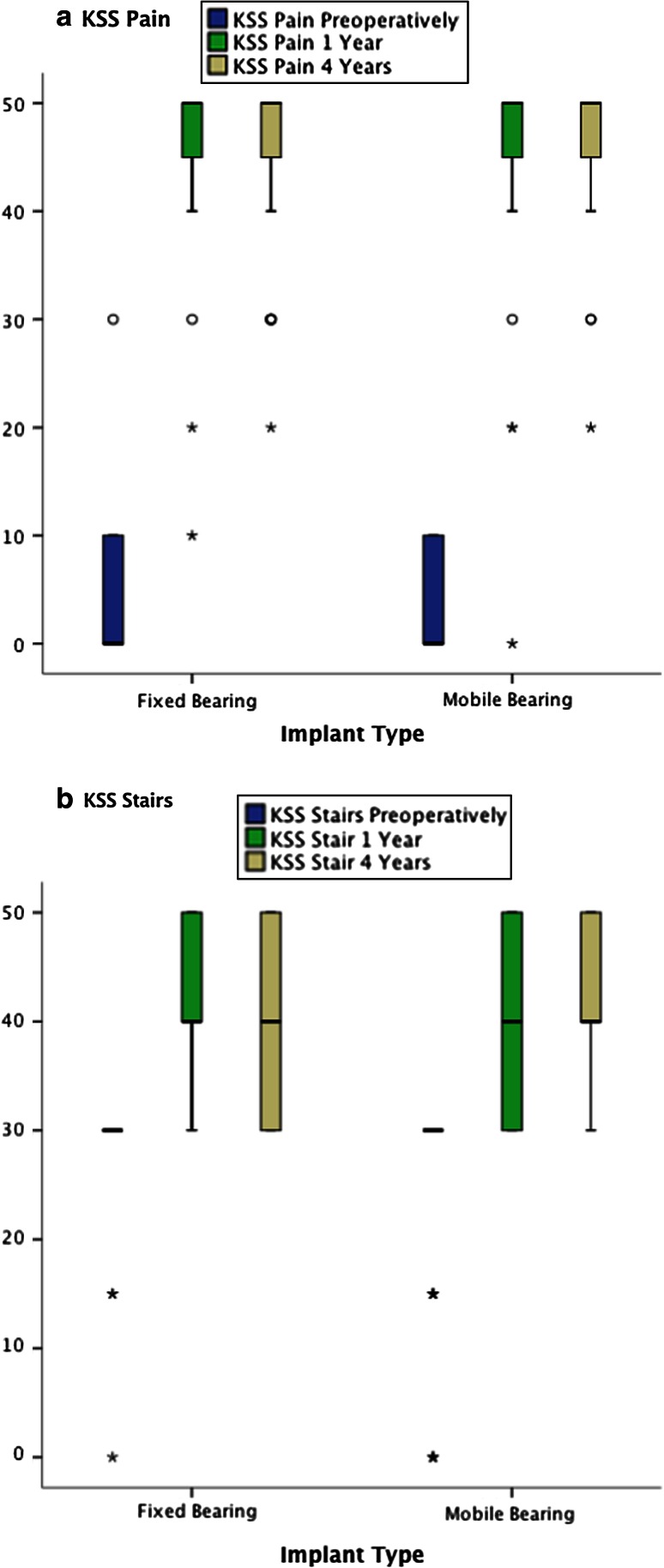
**a** KSS-Pain for the FB and MB groups across the measurement times (pre-operatively and at 1 and 4 years). **b** KSS-Stairs for the FB and MB groups across the measurement times (pre-operatively and at 1 and 4 years)

The median of the sub-item KSS-Pain was the same in both groups across the follow-up times: zero (severe pain) at baseline and 50 (no pain) at 1 and 4 years. There was a significant difference between the mean KSS-Pain ranks (*x* ^2^ = 156.9, *p* < 0.001). The patients perceived a significant pain relieve from pre-operatively to 1 year (*Z* = 8.6; *p* < 0.001). The pain relieve remained at 4 years (*Z* = 0.3; n.s.) (Fig. 2a).

The median of the sub-item KSS-Stairs was equal for both groups across the time: 30 at baseline (up and down with rail) and 40 at 1 and 4 years (normal up, down with rail). There was a significant difference between the mean ranks (*x* ^2^ = 107.3, *p* < 0.001). Patient’s ability to walk stairs improved significantly from pre-operatively to 1 year (*Z* = 7.6; *p* < 0.001) and kept stable from 1 to 4 years (*Z* = 0.8; n.s.) (Fig. 2b).

Intra-operative obtained navigation data on implant positioning, and leg alignment data out of long leg X-rays were similar in both groups without statistically significant differences (Table 4).

**Table 4:** Implant positioning and leg alignment data between the FB and MB groups

Variables	FB	MB	Mean diff.
*Implant positioning* (*navigation data*)			
Femoral angle coronal (FAC)^a^	0.8 ± 0.7	0.8 ± 0.7	0.04 (n.s.)
Femoral angle sagittal (FAS)^a^	0.9 ± 0.6	1.0 ± 0.7	0.05 (n.s.)
Tibial angle coronal (TAC)^a^	0.7 ± 0.4	0.7 ± 0.5	0.01 (n.s.)
Tibial angle sagittal (TAS)^a^	3.7 ± 1.3	3.9 ± 0.9	0.02 (n.s.)
*Leg alignment* (*standing X-ray*)			
Mechanical axis (pre-operatively)^b^	7.8 ± 3.9	8.1 ± 3.5	0.1 (n.s.)
Mechanical axis (post-operatively 1 year)^b^	2.2 ± 1.6	1.7 ± 1.4	0.4 (n.s.)

Values are mean ± SD

*n.s.* non-significant

^a^Deviation (degrees) from 90°

^b^Absolute differences (degrees) from the target alignment

Pearson’s product-moment correlations revealed a week positive relationship between the FAS and the maximal knee flexion at 1 year (*r* = 0.226; *p* < 0.5). There were no significant relationships between the navigation data on implant positioning and the clinical scores (Table 5).

**Table 5:** Relationship between implant positioning, leg alignment data and the clinical scores

Variables	FAC	FAS	TAC	TAS	MA-P	MA-1 Y
KSS-Function 1 year	0.04	0.13	−0.05	0.14	0.03	−0.06
KSS-Knee 1 year	0.12	0.07	0.15	0.09	0.18	−0.18
Maximal Knee Flexion 1 year	0.06	0.22*	−0.09	−0.11	0.04	0.01
Femoral angle coronal (FAC)						
Femoral angle sagittal (FAS)	0.08					
Tibial angle coronal (TAC)	−0.01	−0.06				
Tibial angle sagittal (TAS)	−0.15	0.03	−0.17			
Mechanical axis pre-operatively (MA-P)	0.07	−0.04	−0.09	0.17		
Mechanical axis 1 year (MA-1 Y)	−0.15	0.07	0.21	0.09	0.10	
Mean ± SD	0.85 ± 0.6	0.99 ± 0.6	0.76 ± 0.5	3.8 ± 1.1	8.1 ± 3.7	2.0 ± 1.5

Values are the results of the Pearson’s product-moment correlations (*r*)

* *p* < 0.5

## Discussion

The most important finding of the present study was the lack of results supporting the superiority of one design (FB or MB) over the other in terms of higher ROM, lower pain rates or better patient perceived functional scores. Interestingly, within the first year post-surgery the KSS-Score of the patients in the MB group had a significantly higher improvement rate, though with no differences between the groups neither at 1 nor at 4 years.

The theoretical advantages of MB designs over the conventional FB in TKA were not clinically demonstrated by the time this study was designed. In order to minimize possible influences of different surgical techniques, prosthesis types, expectations of patients and assessors, and post-surgery rehabilitation protocols on the results, a double-blind randomized clinical trial was implemented. Despite dropout rates of 13.5 and 12.5 % in the FB and MB groups, respectively, the sample sizes at 4-year follow-up were still within the preliminarily calculated size of 42 patients per group.

Both groups (FB and MB) were similar at baseline in regard to their demographic data, with exception of “body weight” (*p* = 0.04) with no impact on mean BMI differences (n.s.). There were also no statistically significant differences when comparing the radiological leg alignment data of both groups pre-operatively (Table 4).

The prosthesis type can influence the clinical outcomes as proved in a recent study by Mugnai et al. [24]. In their study, it was demonstrated that the bearing geometry and the kinematic pattern of different prosthetic designs have an effect on clinical outcomes. In the present study, all patients received the same femoral and tibial prosthetic component types and both prosthesis types were posterior cruciate ligament retaining; the only difference between the groups was the mobility of the PE bearing. In addition, all components were cemented. In a recent study [17] on the effects of cemented versus hybrid MB TKA implantations, there were no differences found in terms of revision rates, mortality, alignment deviations or evidence of loosening, when comparing both groups. These results challenge the theoretical assumption that a hybrid fixation (cement-less femoral component) in a MB knee system might increase the rate of loosening of the femoral component.

In a study by Roh et al. [31] comparing highly conforming PCL-retaining vs. PCL-sacrificing MB knees, there were kinematic differences between both procedures, however, with no consequences in terms of significant differences in ROM, functional scores or radiographic results. In the present study, all patients received a PCL-retaining TKA, with no outcome differences when comparing both groups across the time. These results are congruent with the ones by Bailey and colleagues [3].

At baseline, the mean KSS-Function score of the patients in the MB group was ten points inferior (*p* = 0.009) in comparison with the FB group. Despite this significant difference, there were no longer differences when comparing the groups at 1 year, with both groups achieving exactly the same mean KSS-Function score at 4 years. The KSS-Function improvement rate within the first year was significantly greater in the MB group (*p* = 0.01).

The KSS-Knee and Oxford Knee scores of both groups across the time were not significantly different from each other. These results reinforce the ones of recently published randomized controlled trials [1, 3, 5, 12, 22] and are in accordance with the results of lately published meta-analysis [9, 21, 33, 37, 38].

The ROM of both groups was also not significantly different across the time. This result contradicts the one by Aggarwal et al. [1], where the mean ROM was greater in the MB group (*p* = 0.01), but is reinforced by the results of two meta-analysis [9, 21], in which no differences in ROM were found, when comparing FB and MB TKA designs.

Patient’s pain perception was assessed with the use of the KSS questionnaire. At baseline, there was a floor-effect, with the median of both groups situated at “0” points (severe pain) and the second percentile at “10” points (moderate continual pain). TKA reduced pain significantly. At 1 and 4 years, there was a ceiling effect, with the median of both groups placed at 50 points (no pain) and the 1st percentile at 45 points (mild or occasional pain) (Fig. 2a). There were no statistically significant differences when comparing the median or the distribution of values in both groups, showing no advantage of the MB over the FB design in terms of better pain relief. Since the localization of the perceived pain was not discriminated in the present study, it is not possible to answer the question whether the MB design is more patella friendly than the FB design in terms of less anterior knee pain incidence. This may be one of the clinical advantages of the MB with its “self-alignment” of the tibial bearing. In the literature, there are contradictory findings on this matter. In a study by Breugam et al. [6], there were significantly more patients (18.9 %) experiencing anterior knee pain in the FB (posterior-stabilized) then in the MB group (4.3 %) 1 year after TKA. However, the same authors did not confirm their results 7.9 years after surgery [7]. In contrast, a meta-analysis [21] showed lower pain scores in the MB group (OR 0.66; 95 % CI −0.60 to 0.26). Also in a retrospective study by Wyatt et al. [40], there were higher revision rates for resurfacing of the patella in the FB posterior-stabilized TKA than in MB designs, indicating that the implant design may have an influence on the patella-femoral biomechanics.

Computer-assisted TKA allows the surgeon to accurately control parameters related to the implant position and soft tissue balance. In a recent study [14], it was demonstrated that computer-assisted TKA resulted in fewer outliers in frontal leg alignment and tibial component positioning in comparison with conventionally performed TKA. The posterior tibial slope was also better achieved in the computer-assisted group. In the present study, the mean values of the assessed navigation parameters are very near zero, with small ranges and without significant differences in implant positioning when comparing both groups (Table 4). As expected, there were also no significant strong relationships between the implant positioning data and the clinical scores. This result reinforces the ones by Widmer et al. [39]. Also Ishii et al. [18] found no significant correlations between the condylar offset and the maximal knee flexion 1 year post-surgery.

One of the limitations of the present study is the fact that only the golden standard clinical scores were used to compare the groups. Retrospectively, it would have been interesting to have used performance-based standardized tests, like the “Timed Up and Go” or the “Stair Climbing Test” [13, 23, 35], to assess and compare the patients in real-life tasks in a laboratory setting. The OXF is a self-report questionnaire, and the KSS is based on both, patients self-report and assessor’s perception. They are efficient and cost-effective but are they sensitive enough to discriminate functional changes? Patient’s perceptions may not be discriminative enough as shown in a study by Thomsen et al. [36] on the magnitude of knee flexion after TKA, in which the high flexion TKA group achieved significantly higher knee flexion, however, no significant differences in patient’s perceived outcomes were found when comparing the groups. A second limitation of the present study is the fact that patient’s pain perception was assessed according to the KSS protocol without discrimination of pain localization. It would have beneficial to assess pain discrimination with the additional use of the anterior knee pain scale [20].

With the results of this study, the body of evidence supporting no superiority of MB over the FB designs in terms of better functional outcome scores, less pain or higher ROM grows. Further research comparing FB and MB designs should include performance-based measures beside self-reported questionnaires. Furthermore, the assessment of pain should discriminate the pain localization to enable researchers to answer the question whether MB designs are more patella friendly than FB.

## Conclusion

In view of the 4-year results, there is no evidence to support the recommendation of one design over the other in terms of better clinical outcome scores, higher ROM or lower pain rates, since both groups achieved the same outcomes. Long-term follow-up results may be necessary, including survival rates. Further research comparing different TKA designs should also include standardized performance-based tests.
